# Locomotor deficits in recently concussed athletes and matched controls during single and dual-task turning gait: preliminary results

**DOI:** 10.1186/s12984-016-0177-y

**Published:** 2016-07-25

**Authors:** Peter C. Fino, Maury A. Nussbaum, Per Gunnar Brolinson

**Affiliations:** 1 Department of Neurology, Oregon Health & Science University, 3181 SW Sam Jackson Park Road, L226, Portland, OR 97239 USA; 2 Department of Mechanical Engineering, Virginia Tech, Blacksburg, USA; 3 Department of Industrial and Systems Engineering, Virginia Tech, Blacksburg, USA; 4 Edward Via College of Osteopathic Medicine, Blacksburg, USA

**Keywords:** mTBI, Concussion, Brain injury, Gait, Turning, Dual-task

## Abstract

**Background:**

There is growing evidence that mild traumatic brain injury (concussion) can affect locomotor characteristics for prolonged periods of time even when physical signs and symptoms are absent. While most locomotor deficits post-concussion have involved straight walking, turning gait has received little attention despite its pervasiveness in everyday locomotion and athletic competition.

**Methods:**

This study longitudinally examined kinematic characteristics during preplanned turning in a small sample of recently concussed athletes (*n* = 4) and healthy matched control athletes (*n* = 4) to examine potential deficits during single and dual-task turning gait over the initial 6 weeks post-injury, with a one-year follow-up. Turning path kinematics (curvature, obstacle clearance, path length), stride kinematics (stride length, stride width, stride time), and inclination angles were calculated from motion capture of participants walking around an obstacle.

**Results:**

Concussed athletes had larger dual-task costs in turning speed and stride time compared to healthy controls. After controlling for speed and turn curvature, recently concussed athletes increased their inclination towards the inside of the turn over time and decreased their stride time compared to controls indicating a prolonged recovery. Kinematic differences between groups were estimated to recover to healthy levels between 100 and 300 days post-injury, suggesting future prospective longitudinal studies should span 6–12 months post-injury.

**Conclusion:**

Turning gait should be included in future studies of concussion and may be a clinically useful tool. Future longitudinal studies should consider examining gait changes for up to 6–12 months post-injury.

**Electronic supplementary material:**

The online version of this article (doi:10.1186/s12984-016-0177-y) contains supplementary material, which is available to authorized users.

## Background

Sport-related mild traumatic brain injuries, commonly called concussions, affect 1.6–3.8 million people in the United States annually [1]. Within collegiate athletics, concussions account for approximately 5 % of all sport-related injuries [2], making concussion treatment and rehabilitation an important medical concern. While clinical signs and symptoms typically resolve within 10 days [3] there is growing evidence for an “atypical evolution” of symptoms [4] with neurological [5, 6] and locomotor [7–16] changes that persist beyond two months.

The use of a dual-task (DT) paradigm has been particularly successful at identifying residual deficits post-concussion [17]. During DT gait, simultaneous cognitive and motor tasks compete for limited cortical resources [18] and create gait modifications (dual-task costs) compared to single-task (ST) gait. While such dual-task costs (DTC) are present in healthy young adults [19], larger DTCs have been reported in asymptomatic, recently concussed athletes [9–13, 15, 20–24]. The larger DTCs in recently concussed athletes suggest that a concussion may affect the available cortical resources or shift the prioritization from motor to cognitive tasks [18, 21]. In athletics where gameplay, field, and environmental conditions (e.g., crowd noise) create substantial cognitive and physical loads [25], the complexity-dependent motor differences in medically cleared, recently concussed athletes could influence performance and/or injury risk. However, few studies have examined DTCs with complex non-straight movements to simulate the more dynamic demands of competition.

Some studies have reported greater cognitive DTC (cDTC) during obstacle circumvention [11] and hockey-specific skating and puck-handling drills [26], but provided little information about the locomotor DTCs (lmDTC), such as changes in gait speed or mediolateral (ML) sway. Similarly, while several studies have used a step-over obstacle as the secondary task to study lmDTC [9, 27, 28], few studies have combined a complex motor task with a complex cognitive task to elicit larger lmDTCs [10]. No study to our knowledge has yet examined kinematic changes, with or without a cognitive task, in recently concussed athletes during preplanned turning.

Turning gait is a common [29] complex locomotor task [30–33] applicable to change of direction and reorientation during dynamic athletic movements [11, 16]. Yet, despite the high risk of slips and falls [34–36], large COM excursions [37], asymmetrical loading [38], and segmental reorientation variability [16] present during turning, the biomechanical lmDTCs during turning gait in recently concussed athletes remain largely unknown. Due to the high prevalence of turning gait, the segmental reorientation variability in athletes during unplanned turns [16], and the potential correlations to injury risk and performance, both single-task (ST) and DT turning gait in recently concussed athletes present important research areas in concussion management. We hypothesized that ST turning gait would identify kinematic differences between asymptomatic, recently concussed athletes and controls given the increased complexity of turning gait, compared to straight gait, [31–33] and the complexity-dependent effects of concussions on motor performance [10]. The addition of a DT was expected to elicit greater lmDTC during turning in recently concussed athletes compared to healthy controls and further separate concussed and healthy athletes.

## Methods

### Participants

Eight NCAA Division I varsity athletes (four concussed, four matched controls: Table 1) participated in this longitudinal study. Concussions were clinically diagnosed by a trained sports medicine physician and referred to this study by their athletic trainer. No concussed athlete had a prior concussion. For each concussed athlete, a control participant was recruited from teammates of the concussed subject and was individually matched based on sport, position, skill level, and stature. Participants with any history of mental illness, diagnosed cognitive impairment, or unresolved acute lower extremity injury were excluded for the study. Controls were excluded if they had suffered any concussion or brain injury within the past year. No control had any prior history of concussion. All participants gave informed written consent, and all recruitment procedures and experimental protocols were approved by the Virginia Tech Institutional Review Board.

**Table 1:** Descriptive information of all eight participants in matched pairs

ID	Gender	Age (years)	Height (cm)	Mass (kg)	Days Before Full RTP	Weeks Tested^a^
C1 H1	Female	18 19	170 178	63.3 64.6	7 -	(2, 3, 4, 5, 6, 52) (2, 3, 4, 5, 6, 52)
C2 H2	Male	20 20	178 178	78.9 72.4	14 -	(2, 3, 4, 5, 6) (3, 4, 5, 6)
C3 H3	Female	19 18	163 163	58.7 59.0	11 -	(3, 4, 5, 6, 52) (3, 4, 5, 6, 52)
C4 H4	Female	19 21	163 170	59.4 66.6	16 -	(3, 4, 5, 6, 52) (2, 3, 4, 6)

Full return-to-play (RTP) was identified as the first day the athlete was medically cleared for full athletic participation in practices and/or games

^a^ One year follow-up session represented as 52 weeks post-concussion

### Procedures

Participants were tested at approximately weekly intervals for six weeks, at mean (SD) 7 (0), 16 (2), 23 (2), 29 (1), 37 (2), and 45 (3) days following the concussed participant’s concussion. To control for potential musculoskeletal afflictions from recent games or practices that might have influenced gait, each control participant was also tested up to six times and, when possible, on the same days as their competition. Three concussed participants and two control participants also participated in a one-year follow-up testing session occurring at a mean (SD) of 363 (42) days post-concussion. Because of scheduling conflicts, graduations, and transfers, not all participants completed all sessions (Table 1). For the purpose of the current analysis, which examined locomotor changes that remain unresolved after RTP, testing sessions that occurred before the concussed athletes were cleared for full athletic competition were excluded.

**Fig. 1:**
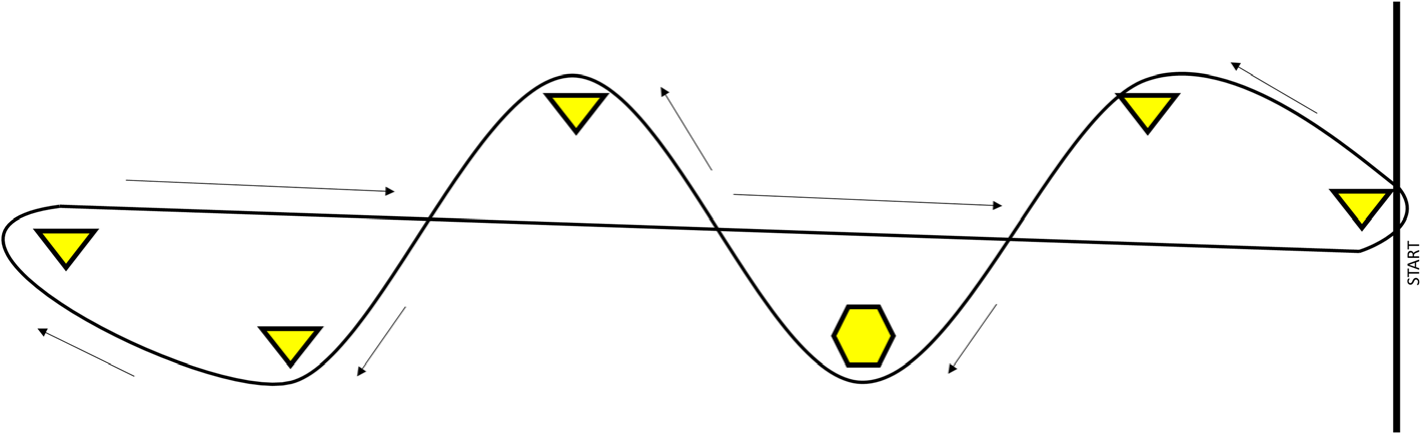
Depiction of the 18 m long course. Triangles indicate pylons. The hexagon indicates the pylon recorded with motion capture cameras

All testing sessions occurred in a basketball gymnasium with minimal distractions and clean hardwood floors. Participants were asked to walk barefoot around an 18 m x 3.5 m course marked with 1.5 m tall pylons (2.5 cm diameter PVC pipe) and consisting of several turns of ~90° (Fig. 1). Participants completed seven laps in each direction around the course. For DT gait, participants were asked to serially subtract by sevens, starting from a random number between 900 and 999, while walking around the course. Participants were not given explicit instruction to prioritize the cognitive or motor task, being instructed only to serially subtract “as fast as you can”. For both ST and DT gait, the participants were instructed not to count the number of laps.

Four motion capture cameras (ProReflex MCU 170 120, Qualisys Medical AB, Gothenburg, Sweden) were stationed around one ~90° turn on the course (Fig. 1) to capture turning kinematics. Reflective spherical markers were placed bilaterally on the calcaneus, with additional markers placed over the xiphoid process and the T9 vertebra. One marker was placed on the pylon to mark its position in space. Data were collected at 120 Hz and filtered using a phaseless fourth-order Butterworth filter with a 6 Hz cutoff frequency. A total of 14 turns, seven of both right and left turns, were captured for each condition corresponding to the 14 laps. Short durations with missing markers were spline interpolated. Trials with more than 30 consecutive missing frames (>0.25 s) were discarded; 30 out of 1120 total trials were discarded.

### Analysis

**Fig. 2:**
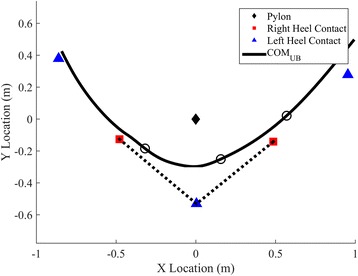
Example overhead view of the turning stride (*dotted line*) connecting the right-left-right heel contacts around the apex during a right turn. The movement tracks right to left. Black circles show the location of the COM_UB_ at each heel contact. The center of mass remains outside of the base of support throughout the entire duration of the turn

The upper-body center of mass (COM_UB_) was estimated using the three-dimensional mean location of the xiphoid process and T9 markers [39]. COM clearance was represented as the minimum horizontal distance from the COM_UB_ to the pylon (*d*_*min*_). The length of the horizontal trajectory, *L*_*path*_, was computed starting when the athlete proceeded below the pylon in the *y* direction, and ending when the athlete advanced above the pylon in the *y* direction (Fig. 2). Quadratic fits to the horizontal trajectory of the COM_UB_ were applied to a moving window of three consecutive points. Subsequently, instantaneous COM_UB_ curvature in the horizontal plane was calculated from the second derivative of each quadratic polynomial fits.

**Fig. 3:**
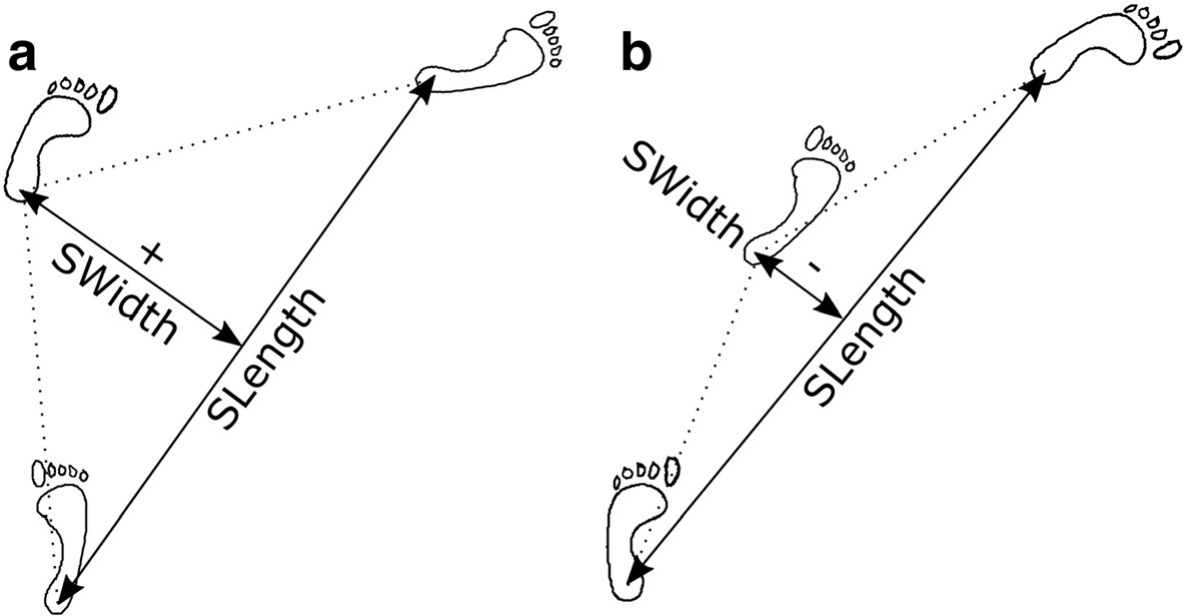
Illustration of step (**a**) and spin (**b**) turns to the right. The stride length, SLength, is the linear distance following the path of progression between successive heel contacts of the same limb. The stride width, SWidth, is the distance, normal to the path of progression, between the apex heel contact and the path of progression

Heel contacts corresponding to each step were identified using the local minima of the heel marker height. For each turn, a single stride was identified that encompassed the point of maximum curvature and therefore the greatest change in direction. This turning stride was comprised of three heel contacts and it was selected such that the middle heel contact (apex limb) occurred closest to the point of maximum COM_UB_ curvature in the *x* direction (Fig. 2). Stride length (SLength) and stride width (SWidth) were calculated based on the line of progression [40], as shown in Fig. 3, and stride time (STime) was calculated as the time from heel contact to heel contact on the same foot within the turning stride. Stride width was allowed to be negative if the apex limb crossed over the line of progression (Fig. 3).

The strategy used within each turn was characterized using the apex limb. A “step turn” was identified if the apex limb was contralateral to the turn direction (e.g., a right turn with the left leg in stance at the apex), whereas a “spin turn” was identified if the apex limb was ipsilateral to the turn direction (e.g., a right turn with the right leg in stance at the apex) [38]. The mean velocity of the COM_UB_ (*v*_*com*_) was calculated over the turning stride. The mean curvature, *k*, of the COM_UB_ was calculated as the mean of the previously described instantaneous curvature over the turning stride to compare the relationship between velocity and curvature.

#### Curvature – velocity power law relationship

A power law relationship between the radius of curvature (*R =* 1*/k*) and velocity has previously been defined for turning gait in both outlined [41] and free paths [42]. This power law relationship can be expressed linearly in logarithmic form

1 $$ \log {v}_{com}(t)= \log A+\alpha \log R(t) $$

where *R* is the radius of curvature, *α* is a constant coefficient, and *A* is the piecewise velocity gain factor that can vary with shape [43]. To compare whether the recently concussed athletes exhibited a different relationship between velocity and curvature, the relationships between mean velocities, *v*_*com*_, and corresponding radii of curvature, 1/*k*, were compared for each group using the *α* and *A* coefficients determined from linear statistical models.

#### Mediolateral inclination angles and centripetal force

**Fig. 4:**
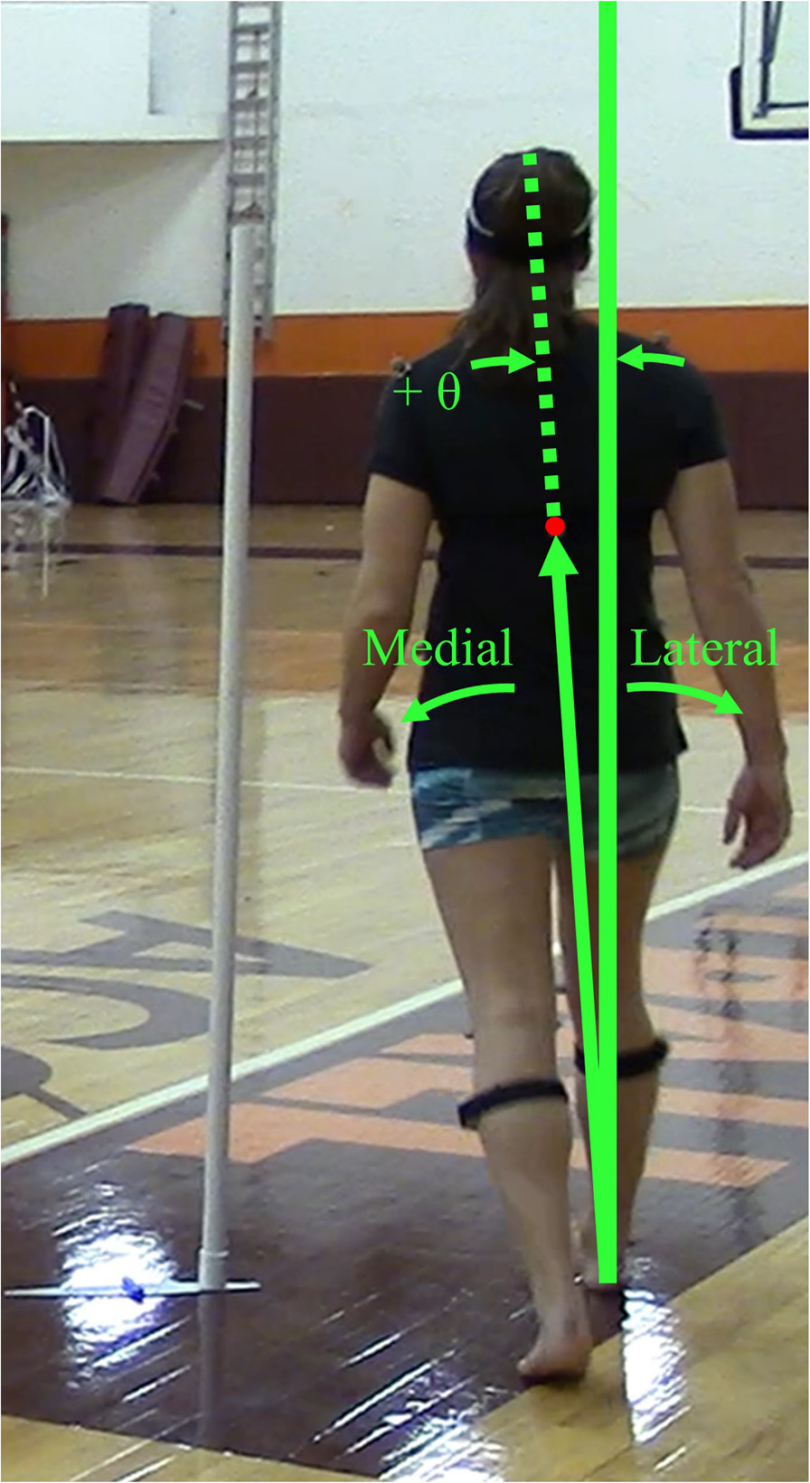
Depiction of the ML inclination angles, with medial inclinations presented as positive and lateral inclinations presented as negative values, regardless of stance limb

The ML inclination angles, *θ*_*1*_, *θ*_*2*_, and *θ*_*3*_, corresponding to the three heel contacts in each turning stride, were estimated using the frontal plane angle between vertical and the vector connecting the heel to COM_UB_ at each heel contact. Because turning is a transient motion, the frontal plane was identified using the vertical plane normal to the instantaneous horizontal velocity of the COM_UB_ [44]. Medial inclinations were defined positive (Fig. 4).

**Fig. 5:**
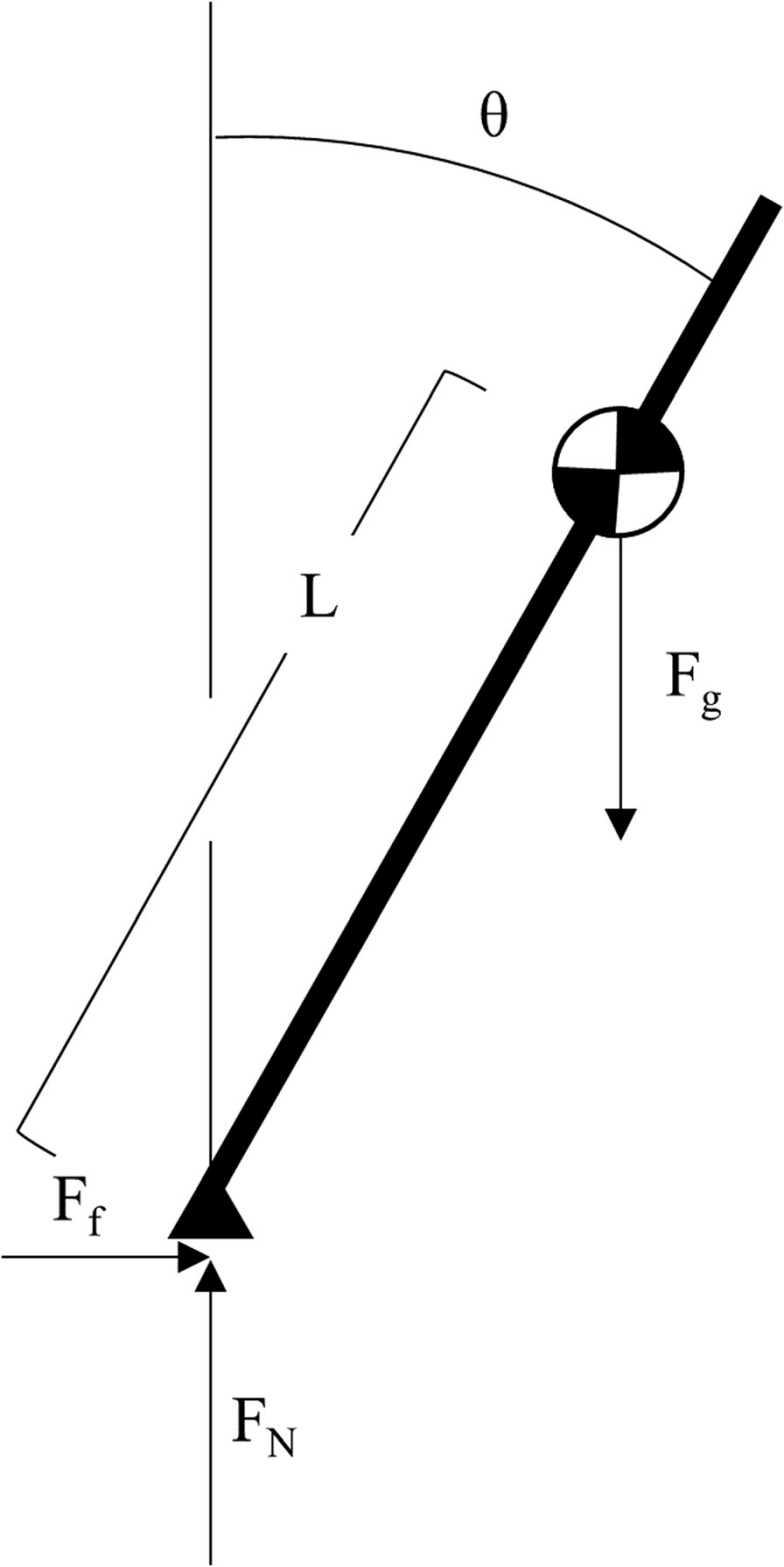
Simple banked rigid body with inclination angle θ, mass m, and length to center-of-mass L

The relationship between inclination angle, velocity *v*_*com*_, and curvature *k* during turning was accounted for using the relation between angle and coefficient of friction, *μ* [37]. Using a simple model of a steady state turn of a banked object (mass *m*) at an angle of *θ* (Fig. 5), balancing centripetal and gravitational moments, *M*_c_ and *M*_*g*_, about the center of mass

2 $$ {M}_c={M}_g $$

3 $$ {F}_f\ L\  \cos \left(\theta \right)={F}_N\ L\  \sin \left(\theta \right) $$

4 $$ \mu\ m\ g \cos \left(\theta \right)=m\ g\  \sin \left(\theta \right) $$

5 $$ \mu = \tan \left(\theta \right) $$

and substituting for *μ* based on the centripetal force relationship

6 $$ {F}_f=m\ {v}^2\ \kappa =\mu\ m\ g $$

7 $$ \mu =\frac{v^2\kappa }{g} $$

the inclination angles follow the proportional relationship

8 $$ \theta \propto { \tan}^{-1}\left({v}^2\kappa \right) $$

where *g* is the gravitational constant. Though variability is introduced from the discontinuities of steps during human locomotion and the transient nature of the turns examined here, this proportional relationship was considered when comparing inclination angles.

A total of 10 kinematic measures were analyzed (Table 2). Because many kinematic outcomes depend on the stance limb and gait speed [37], specific lmDTCs for each outcome were not calculated. Instead, lmDTCs were assessed in the statistical models (see below), with group differences in lmDTC identified by significant group*task interaction effects.

**Table 2:** Description of all measured kinematic outcomes

Outcome	Description
*v*_*com*_	Mean velocity of the COM_UB_ throughout the turn
*k*	Mean curvature of horizontal COM trajectory across turning stride
*L*_*path*_	Total length of COM_UB_ path around the pylon
*d*_*min*_	Minimum distance from the COM_UB_ to the corner pylon
SWidth	Distance from apex limb to the line of progression during the turning stride
SLength	Linear distance from first the third heel contact during the turning stride
STime	Time from first to third heel contact during the turning stride
*θ*_*1*_	ML inclination angle at the first heel contact of the turning stride
*θ*_*2*_	ML inclination angle at the second heel contact of the turning stride
*θ*_*3*_	ML inclination angle at the third heel contact of the turning stride

### Statistical analysis

To assess differences between groups, linear mixed models with random intercepts and slopes for time, which account for the within-subject correlations among each participant’s trials over time, were fit for each outcome. Given previous findings of decreased gait speed during DT gait and in concussed athletes [10], an initial mixed model was evaluated to compare the influence of group, day, task, as well as all interactions on *v*_*com*_. Subsequent models for *k*, *L*_*path*_, and *d*_*min*_, included *v*_*com*_ as a covariate, to account for its influence on the kinematic variables during turning [37]. Outcomes of SWidth, SLength, and STime were stratified by turning strategy to account for the kinematic and kinetic differences between step and spin turns [38]. Two-way interactions of group*day, group*task, and day*task, and the three-way group*day*task interaction were included in the initial models. Interaction terms with *p* < 0.10 were retained in the final models. Kinematic lmDTC differences between groups were reflected in the group*task or group*day*task interaction.

Power law relationships between log *v*_*com*_ and log 1/*k* (Eq. 1) were compared between groups using a mixed model including group and a group*1/*k* interaction term. The exponent *α* and coefficient *A* were determined using the mixed model estimates for each group. Data from concussed one-year follow-ups were excluded from this comparison to prevent potential distortion of the immediate post-concussion relationship.

The inclination angles *θ*_*1*_, *θ*_*2*_, and *θ*_*3*_ were stratified by turning strategy and compared using mixed models with covariates of group, day, task, and tan^−1^(*v*_*com*_^*2*^*k*), following from Eq. 8. Two way interactions of group*day, group*task, day*task, and the three-way interaction of group*day*task were included in initial models. Interactions with *p* < 0.10 were retained in the final models.

If significant group*day interaction effects were present in any of the final models, a linear recovery timeline was constructed using the predicted values from the mixed model. The time from injury to full recovery was then estimated using the time when the model estimates of the concussed and control groups crossed. For all models, assumptions were validated using residuals. All statistical analyses were performed in SAS 9.4 (SAS Institute Inc., Cary, NC, USA) using a two-tailed 0.05 significance level.

## Results

**Fig. 6:**
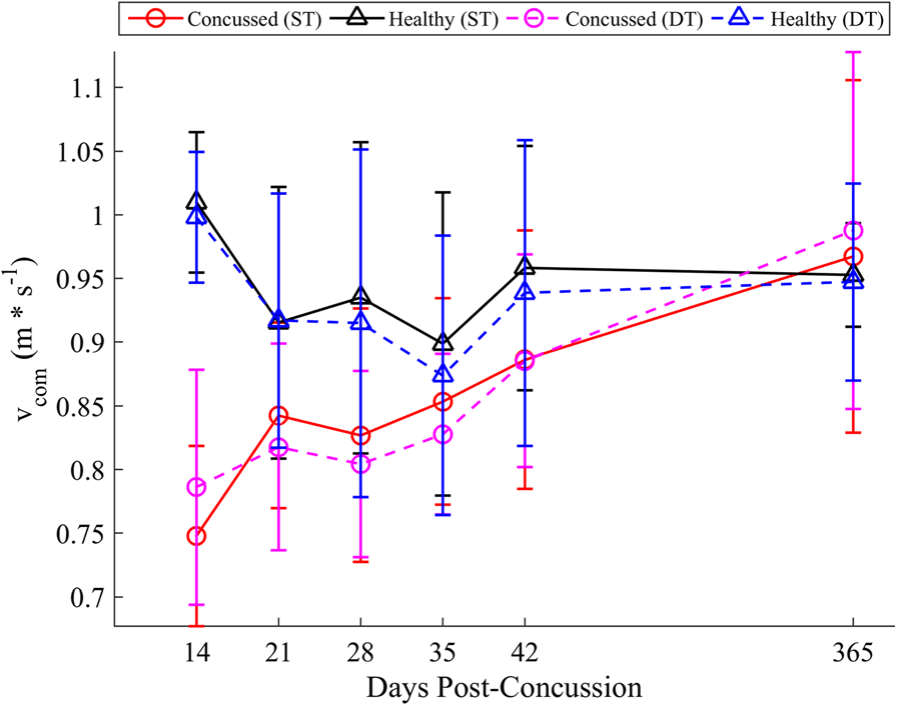
Longitudinal univariate means (SD) of v_com_ for each week. Concussed data are shown in red (ST) and magenta (DT) circles. Healthy controls are shown in black (ST) and blue (DT) triangles

**Fig. 7:**
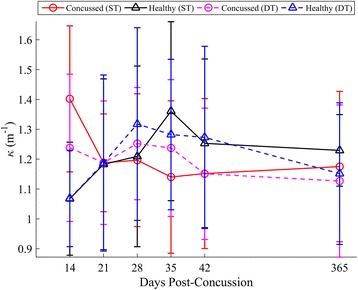
Longitudinal univariate means (SD) of k for each week. Concussed data are shown in red (ST) and magenta (DT) circles. Healthy controls are shown in black (ST) and blue (DT) triangles

**Fig. 8:**
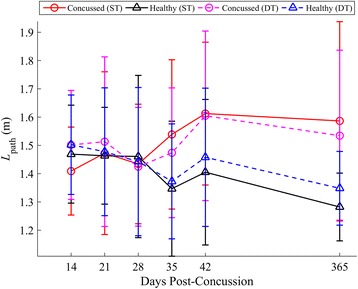
Longitudinal univariate means (SD) of L_path_ for each week. Concussed data are shown in red (ST) and magenta (DT) circles. Healthy controls are shown in black (ST) and blue (DT) triangles

**Fig. 9:**
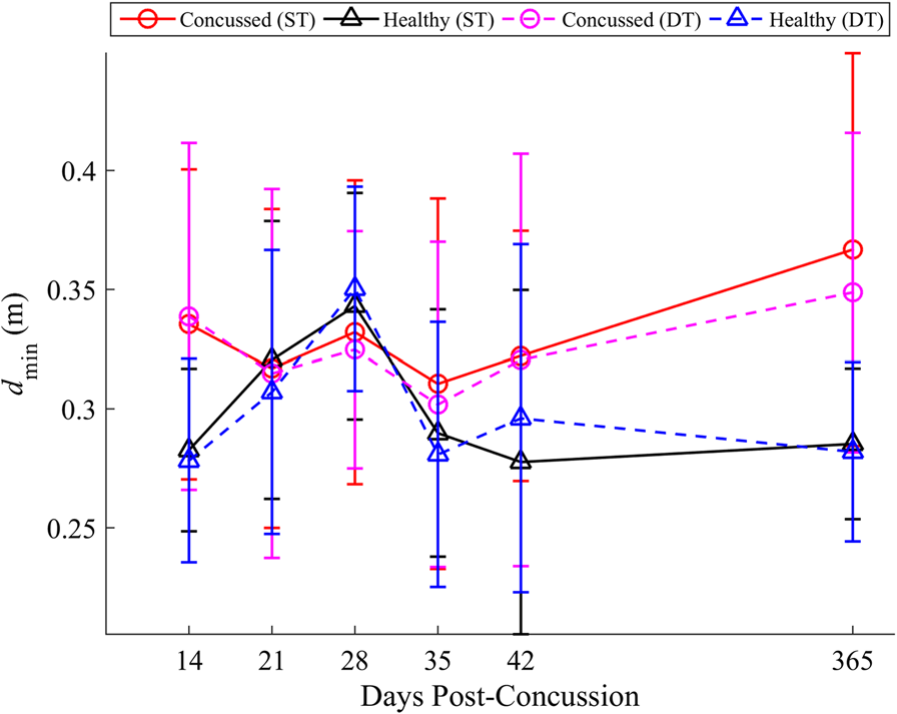
Longitudinal univariate means (SD) of d_min_ for each week. Concussed data are shown in red (ST) and magenta (DT) circles. Healthy controls are shown in black (ST) and blue (DT) triangles

Concussed athletes utilized spin turns at similar frequencies (45 %) to controls (46 %) during both ST and DT turning. A summary of statistical results for *v*_*com*_, *k*, *L*_*path*_, *d*_*min*_ is given in Table 3, with respective longitudinal results in Figs. 6, 7, 8 and 9, respectively. When adjusting for turning velocity, a significant group*day interaction for *k* was found; the concussed group increased their curvature over time, while controls maintained a constant curvature over time. Path length (*L*_*path*_) and COM clearance (*d*_*min*_) did not differ between groups when adjusting for turning velocity. The group*task*day interaction was significant for *v*_*com*_, as the concussed group slowed more than the control group during the DT; the associated lmDTC increased over time (greater difference between ST and DT over time) in the concussed group. Using the mixed model results, the estimated linear recovery time until concussed athletes returned to healthy curvature levels was 398 days.

**Table 3:** Model parameters and inference values from the final linear mixed models for *v*_*com*_, *k*, *L*_*path*_, and *d*_*min*_

	*v*_*com*_		*k*		*L*_*path*_		*d*_*min*_	
	β (SE) *10^3^	*p* value	β (SE) *10^3^	*p* value	β (SE) *10^3^	*p* value	β (SE) *10^3^	*p* value
Group (G)	−70.0 (79.5)	0.379	−139.2 (90.5)	0.124	105.2 (130.6)	0.421	11.1 (37.9)	0.770
Day (D)	0.80 (0.62)	0.243	0.05 (0.13)	0.736	**−0.28 (0.07)**	**0.004**	0.04 (0.04)	0.394
Task Condition (T)	**−28.3 (6.86)**	**<0.0001**	**−22.9 (10.5)**	**0.030**	11.4 (9.6)	0.234	−11.1 (3.47)	0.183
*v*_*com*_	–	–	**−1292 (73.3)**	**<0.0001**	**876.5 (66.1)**	**<0.0001**	**−32.4 (24.3)**	**0.001**
G x D	0.33 (0.86)	0.703	**0.35 (0.17)**	**0.036**	–	–	–	–
G x T	−17.8 (9.51)	0.061	–	–	–	–	–	–
D x T	−0.07 (0.05)	0.198	–	–	–	–	–	–
G x D x T	**−0.21 (0.07)**	**0.003**	**–**	**–**	**–**	**–**	**–**	**–**

Beta coefficients and standard errors (SE) are shown multiplied by 10^3^. Higher-order interactions terms with *p* < 0.10 were retained in the final model. Significant terms are highlighted in bold

**Fig. 10:**
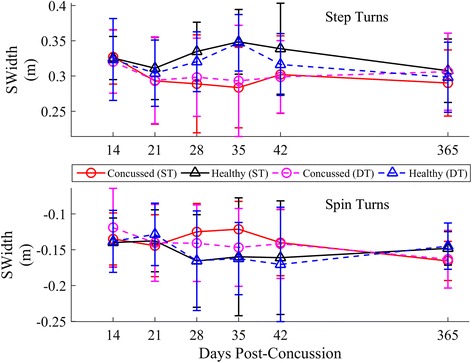
Longitudinal univariate means (.SD) of stride width, SWidth for each week stratified by step (*top*) and spin (*bottom*) turns. Concussed data are shown in red (ST) and magenta (DT) circles. Healthy controls are shown in black (ST) and blue (DT) triangles

**Fig. 11:**
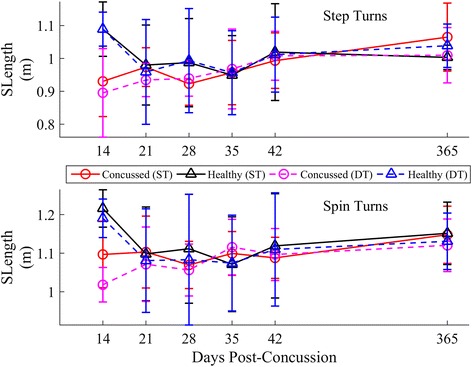
Longitudinal univariate means (SD) of stride length, SLength for each week stratified by step (*top*) and spin (*bottom*) turns. Concussed data are shown in red (ST) and magenta (DT) circles. Healthy controls are shown in black (ST) and blue (DT) triangles

**Fig. 12:**
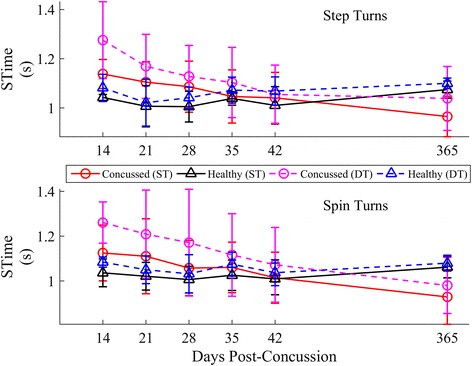
Longitudinal univariate means (SD) of stride time, STime for each week stratified by step (*top*) and spin (*bottom*) turns. Concussed data are shown in red (ST) and magenta (DT) circles. Healthy controls are shown in black (ST) and blue (DT) triangles

Longitudinal results for SWidth, SLength, and STime, stratified by strategy, are provided in Figs. 10, 11 and 12. Significant lmDTCs, indicated by significant group*task interactions, were found for STime during both step and spin turns and for SWidth during step turns (Table 4). Significant group*day interactions indicated that the concussed group recovered toward healthy levels over time in SWidth for step turns, STime for step turns, and STime for spin turns. The estimated time until concussed athletes recovered to healthy turning stride characteristics during step turns, assuming a linear recovery, was 391 and 144 days for SWidth and STime, respectively, and 179 days for STime during spin turns. STime increased during the DT condition regardless of strategy. Though SLength increased over time (step turns) and decreased during DT turning (spin turns), no differences in SLength were found between groups.

**Table 4:** Model parameters and inference values from the final linear mixed models for SWidth, SLength, and STime

	SWidth		SLength		STime	
	β (SE) *10^3^	*p* value	β (SE) *10^3^	*p* value	β (SE) *10^3^	*p* value
Step (*n* = 593)						
G	−42.9 (33.5)	0.201	−31.1 (71.6)	0.664	51.5 (57.8)	0.373
D	−0.05 (0.02)	0.088	**0.27 (0.10)**	**0.035**	0.11 (0.07)	0.193
T	−7.7 (4.3)	0.076	−6.7 (6.3)	0.296	**33.1 (9.1)**	**0.0003**
G x D	**0.11 (0.03)**	**0.0002**	–	–	**−0.36 (0.10)**	**0.0002**
G x T	**12.5 (5.9)**	**0.034**	–	–	**37.5 (12.3)**	**0.003**
Spin (*n* =497)						
G	15.6 (26.6)	0.556	−29.5 (77.0)	0.702	67.8 (73.2)	0.355
D	**−0.06 (0.02)**	**0.012**	0.47 (0.23)	0.085	0.07 (0.07)	0.341
T	−1.5 (3.9)	0.701	**−18.0 (5.6)**	**0.001**	**32.2 (11.8)**	**0.007**
G x D	–	–	–	–	**−0.38 (0.09)**	**<0.0001**
G x T	–	–	**–**	**–**	**38.5 (16.4)**	**0.023**

Beta coefficients and standard errors (SE) are shown multiplied by 10^3^. Higher-order interactions terms with *p* < 0.10 were retained in the final model. Each outcome was stratified by turning strategy. Significant terms are highlighted in bold

**Fig. 13:**
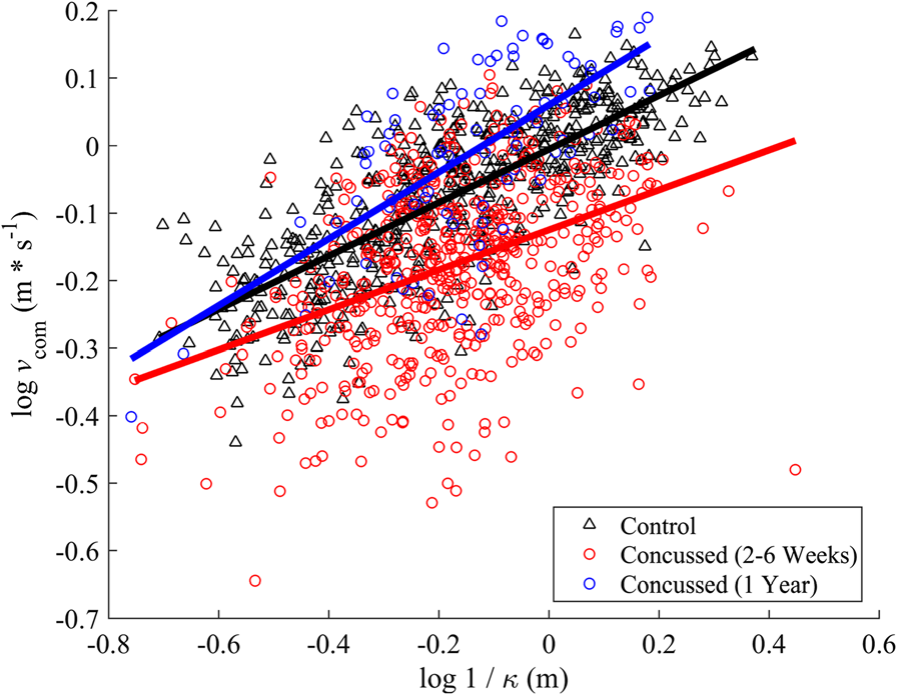
Linear fits of the power law relationship between velocity and radius of curvature for controls (*black*, *R* ^2^ = 0.59), concussed athletes within 6 weeks on injury (red, *R* ^2^ = 0.18) and concussed athletes after one year (*blue*, *R* ^2^ = 0.45). No difference in slopes were found between the fits, but a significant decrease in the velocity gain function A in concussed athletes 2–6 weeks post-concussion was found

Linear fits between log *v*_*com*_ and log 1/*k* yielded estimated mean (SE) of −0.24 (0.06) for parameter *A* in concussed athletes within 6 weeks of their concussion, and −0.08 (0.04) in controls (Fig. 13). The difference between groups was significant (*p* = 0.009), indicating that *A* significantly decreased in concussed athletes during the first 6 weeks. No significant interaction was found, indicating that *α* was similar between concussed and controls, whose respective values were 0.24 (0.03) and 0.21 (0.02).

**Fig. 14:**
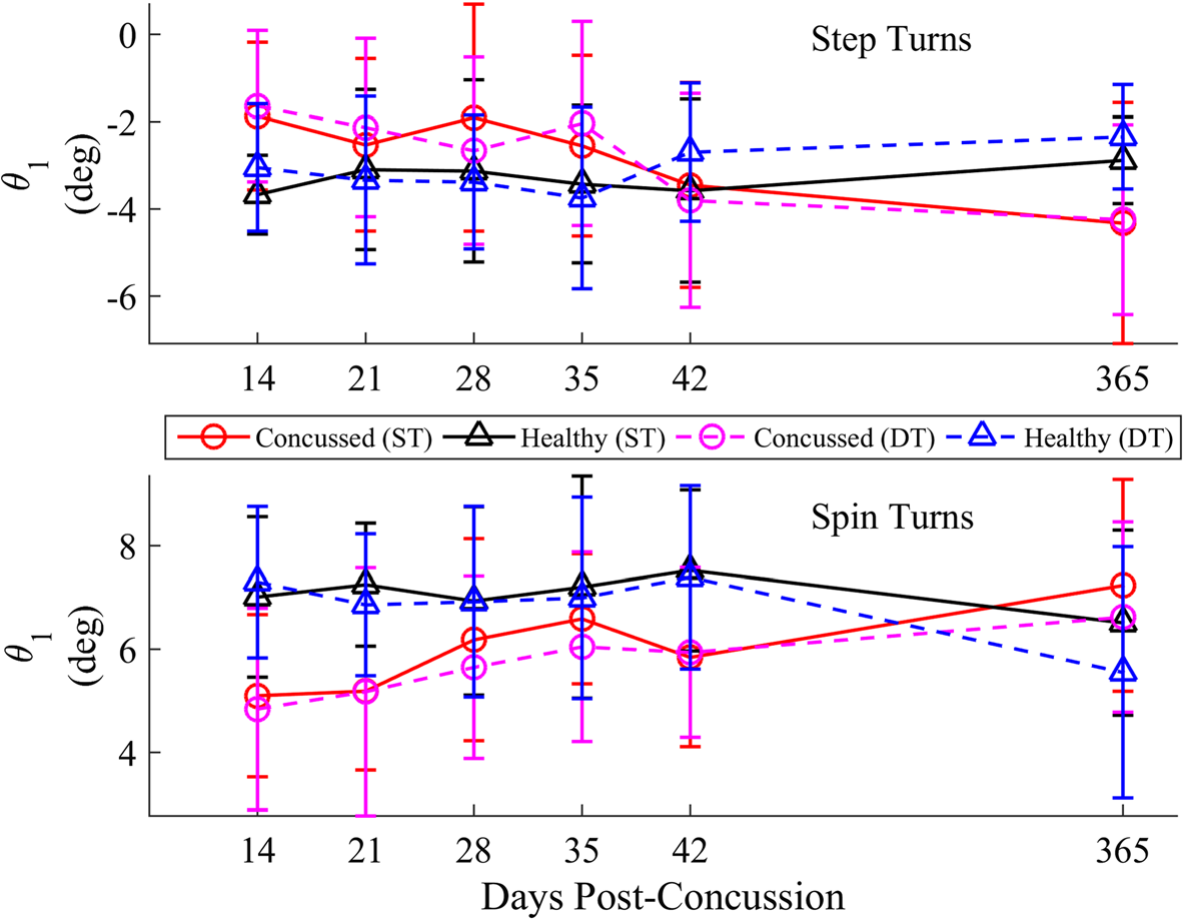
Longitudinal univariate means (SD) of ML inclination angle θ_1_ for each week stratified by step (*top*) and spin (*bottom*) turns. Concussed data are shown in red (ST) and magenta (DT) circles. Healthy controls are shown in black (ST) and blue (DT) triangles

**Fig. 15:**
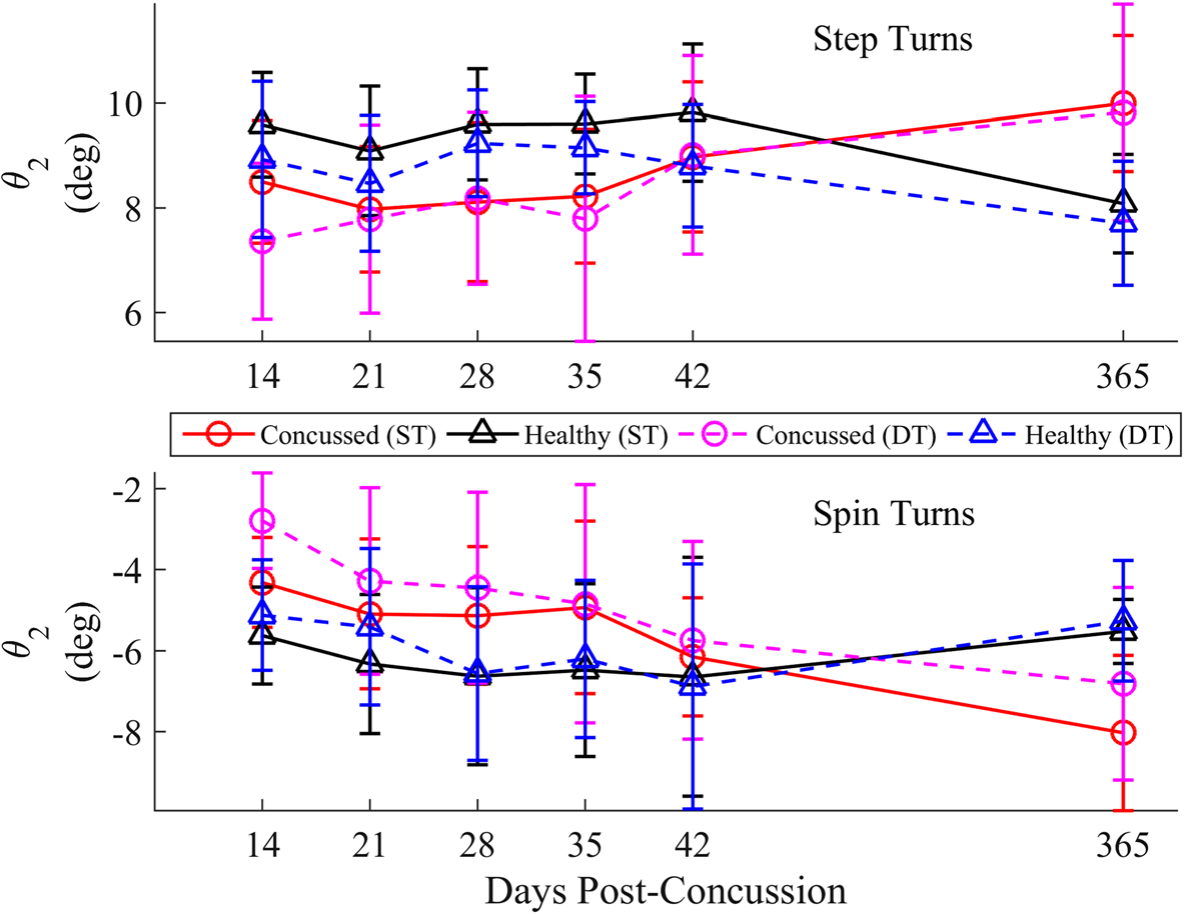
Longitudinal univariate means (SD) of ML inclination angle θ_2_ for each week stratified by step (*top*) and spin (*bottom*) turns. Concussed data are shown in red (ST) and magenta (DT) circles. Healthy controls are shown in black (ST) and blue (DT) triangles

**Fig. 16:**
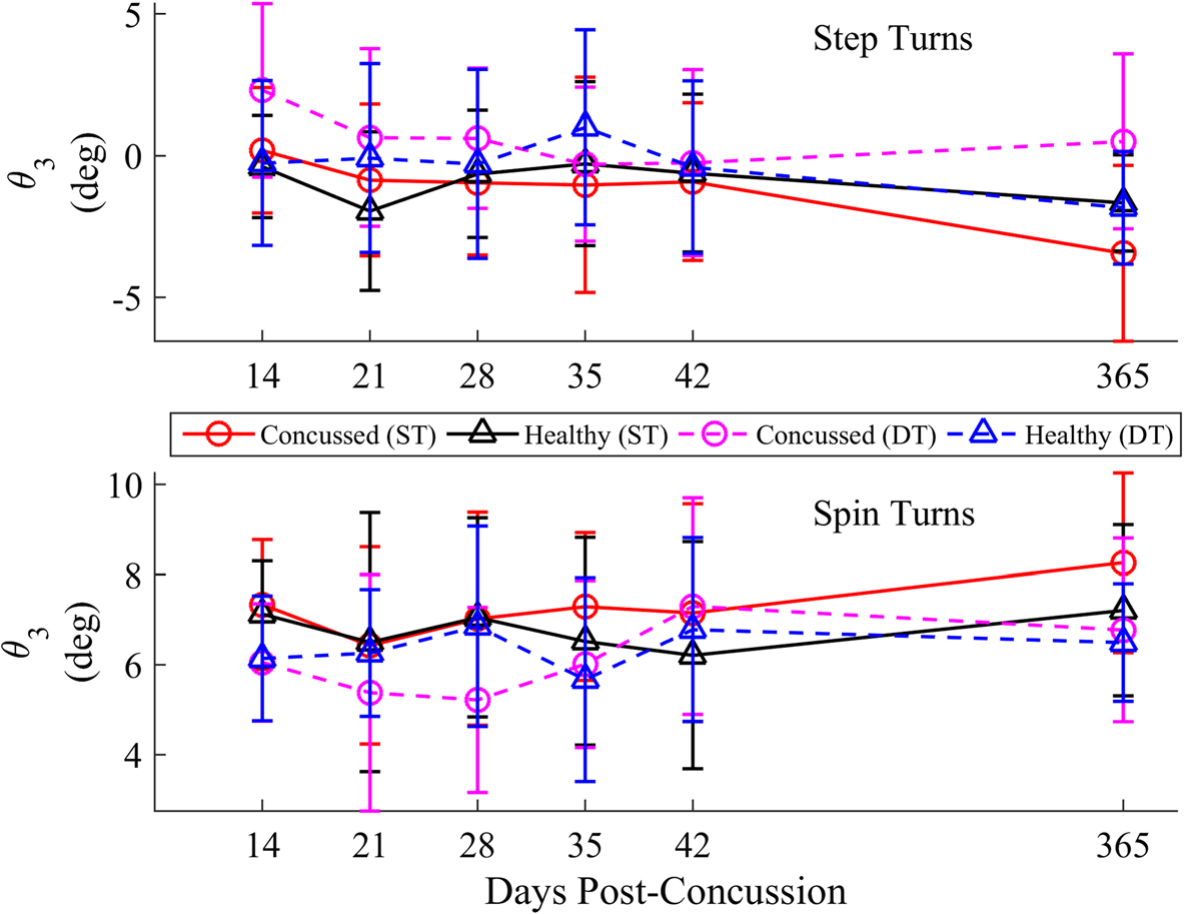
Longitudinal univariate means (SD) of ML inclination angle θ_3_ for each week stratified by step (*top*) and spin (*bottom*) turns. Concussed data are shown in red (ST) and magenta (DT) circles. Healthy controls are shown in black (ST) and blue (DT) triangles

Longitudinal results for each angle are provided in Figs. 14, 15 and 16. When adjusting for the tan^*−*1^(*v*_*com*_^*2*^*k*) term (Table 5), significant group*day interactions were found for *θ*_*1*_ during spin turns and *θ*_*2*_ for both strategies, indicating that the concussed group increased the magnitude of inclination with increasing time post-injury. For ipsilateral stance limbs during the second half of the turning stride (*θ*_*3*_ step turns), a group*day*task interaction was found, indicating an *increase* in the lmDTC difference between concussed and control groups as time increased. The estimated linear recovery time for concussed athletes was 115 and 123 days during step turns for *θ*_*2*_ and *θ*_*3*_, respectively, 239 days for *θ*_*1*_ during spin turns, and 222 days for *θ*_*2*_ during spin turns. As the DTC differences increased for *θ*_*3*_ over time, no recovery date could be estimated.

**Table 5:** Model parameters and inference values from the final linear mixed models for the three ML inclination angles, θ_1_, θ_2_, and θ_3_

	*θ*_*1*_		*θ*_*2*_		*θ*_*3*_	
	β (SE) *10^3^	*p* value	β (SE) *10^3^	*p* value	β (SE) *10^3^	*p* value
Step (*n* = 593)						
G	151.0 (653.6)	0.817	−677.7 (623.6)	0.278	806.0 (1056)	0.446
D	−0.09 (0.74)	0.904	−2.2 (1.8)	0.275	−3.2 (2.5)	0.236
tan^*−*1^(*v*_*com*_^*2*^ *k*)	**−7241 (899.0)**	**<0.0001**	**4605 (565.3)**	**<0.0001**	**6754 (1441)**	**<0.0001**
T	−94.6 (149.4)	0.527	**−291.2 (90.0)**	**0.001**	**1198 (394.9)**	**0.003**
G x D	–	–	**5.9 (2.4)**	**0.015**	**−6.6 (3.2)**	**0.042**
G x T	–	–	**–**	**–**	203.0 (542.1)	0.708
D x T	–	–	**–**	**–**	−3.1 (3.0)	0.295
G x D x T	–	–	**–**	**–**	**9.8 (4.0)**	**0.016**
Spin (*n* = 497)						
G	**−1338 (456.2)**	**0.004**	1107 (1241)	0.373	443.5 (474.8)	0.351
D	−2.7 (1.7)	0.172	−0.53 (1.33)	0.704	0.24 (0.81)	0.775
tan^*−*1^(*v*_*com*_^*2*^ *k*)	**3738 (1009)**	**0.0002**	**−5344 (769.4)**	**<0.0001**	**7490 (1109)**	**<0.0001**
T	−77.4 (157.9)	0.625	356.2 (194.6)	0.068	**−412.1 (177.0)**	**0.023**
G x D	**5.6 (2.3)**	**0.016**	**−5.0 (1.8)**	**0.005**	–	–
G x T	**–**	**–**	−222.5 (272.1)	0.414	–	–
D x T	**–**	**–**	−1.4 (1.5)	0.370	–	–
G x D x T	**–**	**–**	4.0 (2.1)	0.054	–	–

Beta coefficients and standard errors (SE) are shown multiplied by 10^3^. Higher order interactions terms with *p* < 0.10 were retained in the final model. Outcomes were stratified by turning strategy. Significant terms are highlighted in bold

## Discussion

This study prospectively examined single- and dual-task turning kinematics in recently concussed and healthy control athletes. It provides the first preliminary evidence of kinematic differences during ST and DT preplanned turning gait in a small sample of recently concussed athletes. Despite all athletes being medically cleared for competition, concussed athletes showed greater lmDTCs in SWidth, and STime compared to healthy controls. Additionally, several outcomes – including SWidth, STime, ML inclination angles (*θ*_*1*_, *θ*_*2*_, and *θ*_*3*_) and curvature – showed different patterns over time between groups when adjusted for turning speed, which suggests that turning gait may recover slower than other symptoms. Overall, these differences between groups over time describe the effects of concussion on turning gait in three domains: path trajectory (*k*), stride characteristics (SWidth, SLength, STime), and body orientation (*θ*_*1*_, *θ*_*2*_, *θ*_*3*_).

### Path trajectory

One key finding of this preliminary study is a decreased velocity gain factor (*A*) in concussed athletes relative to healthy controls. While both groups exhibited similar power law relationships between *v*_*com*_ and 1/*k*, the concussed group walked with slower velocities across the range of curvatures. Similar modulations of *A* have been reported as individuals transition to different outlined trajectories [41, 43], but only when the path is predefined. Though individuals follow stereotyped behavior without a predefined path [45], Olivier and Cretual [42] argued that the lack of a consistent power relationship over the course of one turn indicated that a simple turn is not performed by first planning the trajectory and then by following the planned path. Instead, the motor control system is simplified by coupling velocity and curvature and minimizing the deviations in an orientation/velocity phase space from one trajectory to the next [42]. We found similar *α* values between the concussed and control groups, suggesting that this long term coupling remains present post-concussion. However, the lower *A* values post-concussion indicate that the relationship has shifted to a slower speed, consistent with previously described decreases in gait speed post-concussion [10]. Given the similar values of *α* between groups, though, it is possible that some of the navigational deficits reported in concussed individuals [11] are predominantly changes in gait speed and not differences in an intrinsic velocity – curvature relationship that may affect path characteristics. We have previously reported differences in straight gait speed in this sample population [46] matching previously reported data [15]. Turning speed may also be a useful indicator of recovery based on the present results.

**Fig. 17:**
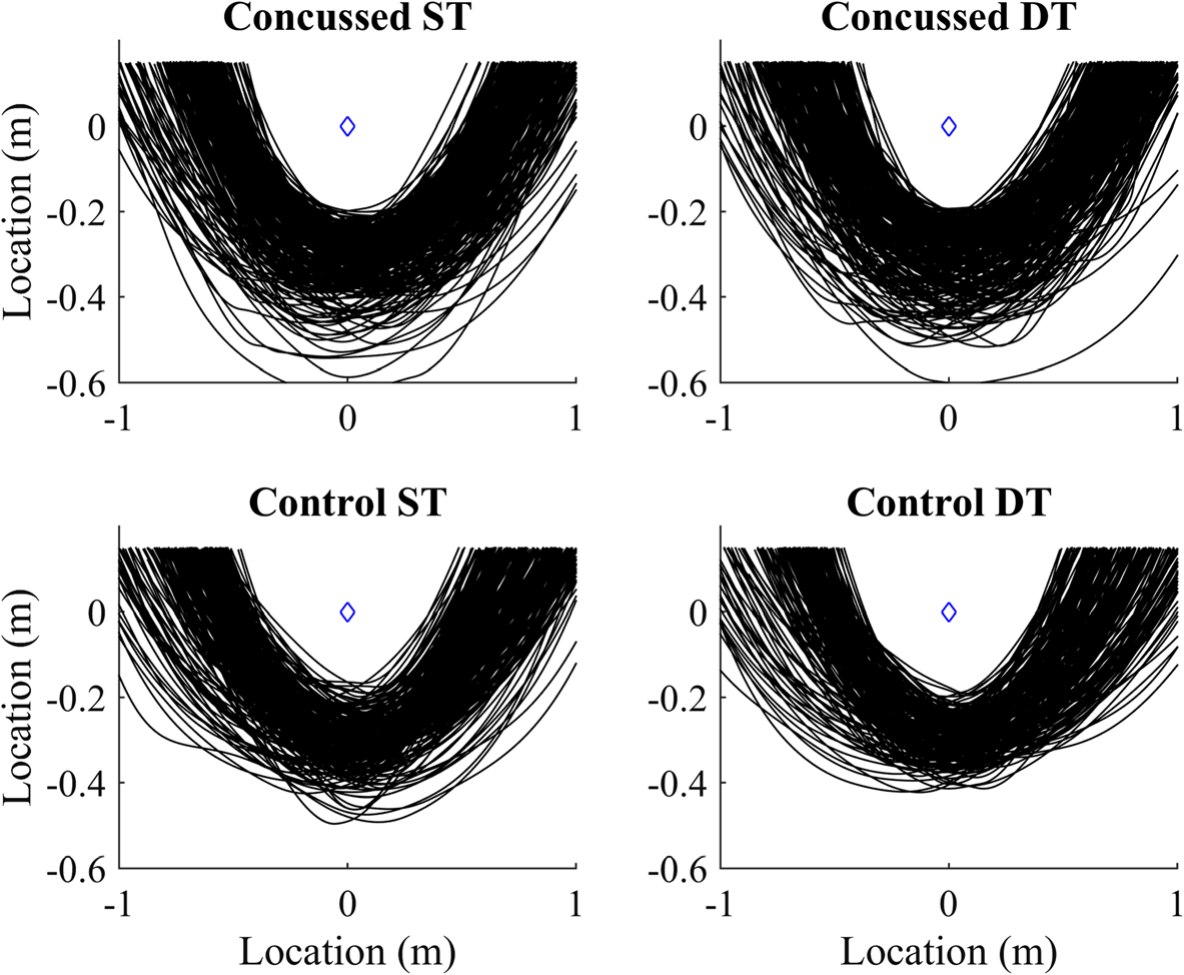
All trajectories for single- (*left*) and dual-task (*right*) turning. The concussed group’s trajectories are shown in the top two figures; the controls on bottom. The pylon is represented with the blue diamond at the origin. All distances are in meters

Our results do not indicate whether the path selection was quantitatively different between groups, but trajectories between groups and conditions were qualitatively similar (Fig. 17) and there were no significant differences in path length (*L*_*path*_) or minimum clearance (*d*_*min*_). The present results, as evidenced by low *R*^2^ values from the velocity-curvature relationship (Fig. 12) in concussed compared to healthy athletes, do agree with Baker and Cinelli [7] that concussed athletes are more variable in their path selection. It is likely that, on average, concussed athletes can exhibit healthy stereotyped path selection [45, 47] but do so with greater variability, similar to their increased joint-coordination [27] and step variability [14].

We did not find a significant difference between groups in minimum clearance (*d*_*min*_), though overall conclusions are limited due to the small sample size. However, this appears to contradict Fait et al. [11], who described greater minimum clearances in athletes post-concussion compared to controls. Upon further investigation, however, the apparent differences in clearance reported by Fait et al. [11] may have been due to slightly slower gait speeds in the concussed group. Results presented here and by Fino et al. [37] showed a strong inverse relationship between walking velocity and the minimum obstacle clearance. Additionally, Fait et al. [11] utilized an in-line obstacle circumvention paradigm, where the goal remained fixed and an obstacle was placed in the direct path to that goal. Conversely, we used an obstacle navigation task, where participants were instructed to pass on a specific side of each obstacle in route to the next. It is possible these different tasks elicited distinct behaviors [48] considering that the required change in heading angle was much larger in the current study.

### Stride characteristics

Concussed athletes exhibited consistent lmDTC increases in SWidth and STime, widening and slowing their strides during the DT more than the control group. This greater slowing of strides during DT concussed gait has previously been described in straight walking [15, 49], but no prior work (to our knowledge) has described this result during turning gait. Larger DTCs in recently concussed athletes are often attributed to a diminished multi-tasking capacity post-concussion, suggesting that a concussion may affect the available cortical resources or shift the prioritization from motor to cognitive tasks [18, 21].

We did not find differences in stride length between groups. Previous studies have reported a decrease or no change in stride length in recently concussed athletes during DT straight walking [23, 49, 50]. Based on the longer STimes in the concussed group and similar SLengths between groups, the differences in gait speed between groups were likely controlled by increasing the STime and not by shortening the SLength during turning.

### Body orientation

Recently concussed athletes increased their inclination towards the turn over time, nearing the inclination angles used by controls, after adjusting for the tan^*−*1^(*v*_*com*_^*2*^*k*). Based on a simplistic model of a continuous rolling rigid body, the inclination angle should be directly proportional to tan^*−*1^(*v*_*com*_^*2*^*k*). Given that the concussed athletes generally walked slower across similar curvatures, lower magnitudes of *θ* in concussed athletes were expected. However, this group difference was present, though it resolved over time, even when adjusting the model for tan^*−*1^(*v*_*com*_^*2*^*k*). For a given velocity and curvature, concussed athletes exhibited less inclination towards the inside of the turn (less medial inclination during step turns, less lateral inclination during spin turns). One may interpret this decreased inclination as another cautious adaptation of the concussed group in order to preserve stability [13, 21, 49] and maintain the COM closer to the base-of-support (BOS). Yet, concussed individuals exhibit greater ML sway during straight walking and obstacle crossing [12, 20, 23, 28, 49]. At this time, it is unclear why this paradoxical change occurs between curved and straight walking.

While only limited kinematic data were collected in this preliminary study, the geometric relationship between inclination angle and centripetal force can be used as an initial step to examine potential kinetic differences between concussed and control athletes during turning. Using the simple banked rigid body model (Fig. 5), a change in *θ* introduces a new coronal plane moment if velocity, curvature, and mass are held constant (based on Eq. 3). For a given curvature and velocity, a decrease in *θ* induces a moment counteracting the moment from centripetal force acting on the COM; an increase in *θ* induces a moment acting with the centripetal force moment. Interestingly, similar placement of the COM closer towards the stance limb during cutting, accomplished through lateral trunk flexion, has been associated with ACL injuries [51], illustrating the potential risk of decreased inclination during sport-related maneuvers. The current preliminary investigation was not designed to investigate how, where (ankle, knee, hip), or if these increased moments manifest. Yet, these initial results encourage future research examining potential kinetic differences during fast-paced sport-related turning and cutting following concussion, which may be a contributing factor to the increased rate of acute musculoskeletal injuries post-concussion [52, 53].

### Timeline of effects and recovery

Notably, all data presented here were collected after the concussed athletes had returned to full athletic participation. Thus, these results support the growing body of literature indicating locomotor capacity has a prolonged recovery compared to clinical symptoms [7–13, 15, 16, 20–24, 26]. Despite the absence of clinical signs or symptoms, gait deficits post-concussion persisted, and it is unclear if or when any such deficits resolve. Several locomotor abnormalities have been found to persist at least 30 days post-concussion [7, 11, 13, 14, 26], with some deficits lasting beyond 60 days [12]. Our results agree with this earlier evidence, by predicting that the recovery of several kinematic abnormalities persist beyond six weeks post-concussion. Yet, we are not aware of any previous study that has prospectively examined recovery beyond 2 months to investigate a full recovery timeline. Approximations based on group*day interaction terms in the present statistical models suggest that complete recovery may not occur until after 200–300 days post-concussion. These linear approximations should be interpreted cautiously, though, given the likelihood of a non-linear recovery pattern, the reliance on only mean estimates, the small sample size, and the general lack of data immediately surrounding the estimated recovery time. However, the results do give guidance for the design of future prospective longitudinal studies examining locomotor capabilities post-concussion, suggesting the need to follow participants for 6–12 months post-concussion. Interestingly, these estimates do fit within the same timeframe of reported increases in musculoskeletal and subsequent injuries post-concussion [52–54], reinforcing the need for comprehensive 6–12 month longitudinal studies.

### Limitations

The small sample size of this preliminary study is a primary limitation and prompts caution when generalizing these results to an entire population. Nevertheless, significantly different lmDTC and changes in kinematic variables over time were found between concussed and healthy controls. Additionally, despite the small sample, this presents the first investigation into turning kinematics post-concussion and provides the only longitudinal data spanning up to one year. Variations in a few data points, such as the one-year follow-up data among controls, likely had particularly strong influence on the recovery timeline, potentially leading to over- or underestimated recovery in some instances (e.g., for *θ*_*2*_ the recovery time may have been underestimated given the decrease in inclinations of the two controls subjects at the one-year follow-up).

Another limitation was the potential variation of cognitive task performance. The serial seven subtraction task was chosen based on its complexity and the ability to discriminate concussed and healthy individuals [23]. While participants were directed to subtract as fast as possible, it was difficult to discern whether priority was given to the cognitive or motor task, as the response rate and number of errors were difficult to characterize. However, because this investigation was concerned with the kinematic deficits during locomotion that manifest in asymptomatic cleared athletes, potential differences in prioritization do not change the present conclusions about diminished dual-task capacity. Similar prioritization would likely occur during real-life situations outside the testing environment.

Finally, the use of a small set of reflective markers may have introduced small error into the COM_UB_ and inclination angle *θ* calculations compared to a full body marker set. Other markers were placed on the participants, but proved unreliable due to the testing environment.

## Conclusions

Turning gait kinematics changed differently over time between concussed and healthy athletes, suggesting that turning locomotion recovers slower than clinical signs or symptoms. These results also suggest that turning velocity and stride time may be useful clinical assessment tools and that turning mechanics post-concussion, reflected in the mediolateral inclination angles during turning gait, may reflect potential kinetic differences during sport-related maneuvers (e.g., cutting) that could contribute to the higher musculoskeletal injury rates post-concussion [52, 54]. Turning gait and change of direction tasks are recommended for inclusion in future locomotor studies of concussion-related kinematic differences and injury potential [16], and that such studies extend for six months to one year post-concussion to capture the full recovery timeline. Overall, these results reaffirm that concussions can present persistent gait modifications, and that when and how such gait disturbances subside should be an important area for future research.

## Abbreviations

*A*, power law gain factor between velocity and radius of curvature; BOS, base of support; C, concussed athletes; cDTC, cognitive dual-task cost; COM, whole-body center of mass; COM_UB_, center of mass of the upper body; DT, dual-task; DTC, dual-task cost; H, healthy matched controls; lmDTC, locomotor dual-task cost; ML, mediolateral; mTBI, mild traumatic brain injury; NCAA, National Collegiate Athletic Association; RTP, return to play; SD, standard deviation; SE, standard error; ST, single-task; *α*, power law exponent between velocity and radius of curvature; *μ*, coefficient of friction

## Additional file

Additional file 1: Dataset used for all analysis. (XLSX 400 kb)
